# Explainable AI based ensemble model for the identification of Schizophrenia prodromal phase

**DOI:** 10.1038/s41598-026-48761-3

**Published:** 2026-04-18

**Authors:** R. Roopalakshmi, Neha J. Rao, Meeti Foram Mehta

**Affiliations:** Manipal Institute of Technology, Manipal Academy of Higher Education (MAHE), Manipal, Karnataka 576104 India

**Keywords:** Schizophrenia detection, Ensemble learning, Explainable AI, SHapley additive values, Machine learning, Feature selection, Schizophrenia, Machine learning, Predictive medicine

## Abstract

Schizophrenia is a psychotic spectrum disorder, which impacts multiple domains, including cognitive functioning, interpersonal relationships, impairments in daily activities and ultimately reduces the quality of life for affected individuals. As a chronic mental health condition, schizophrenia affects millions of people worldwide and leads to cognitive dysfunctions and abnormal behaviors in patients. In recent decades, Artificial Intelligence (AI) has revolutionized healthcare, by making remarkable contributions towards disease diagnosis, personalized treatment planning, and enhanced patient care outcomes. Therefore, we presented an AI framework using Machine Learning (ML) and Ensemble Learning (EL) models along with Feature Selection to predict prodromal symptoms in Schizophrenia patients. We utilized an Open-source dataset collected from 5000 patients comprising clinical, psychological and behavioral symptoms. Among all the developed classifiers, the customized EL-based STACK models achieved best results for Accuracy, Precision, Recall, F1-score and Average AUC of 96.2%, 96%, 96%, 96.7%, and 93% respectively. Recently, Explainable AI (XAI) techniques are gaining attention in making classifier predictions more interpretable, understandable and reliable. Therefore, we employed XAI-based Shapley Additive exPlanations (SHAP) architecture, for generating visualizations including Violin, Waterfall, Force and Dependence Plots, which deliver meaningful interpretations of proposed classifier predictions. The motivation of this research study is to correctly predict the prodromal symptoms of Schizophrenia disease by employing EL-based stacking classifiers integrated with XAI tools, which can help the clinicians in making informed decisions.

## Introduction

Schizophrenia is a major psychiatric illness, which is associated with hallucinations and causes life-threatening disorganized behaviours, cognitive impairments, social disengagements in patients^1,2^. Specifically, around 20 million people across the globe and nearly 1 million individuals in India are currently affected by this schizophrenia disorder^3^. In general, Schizophrenia symptoms are divided into three broad categories: Positive, Negative and Cognitive symptoms respectively^4^. The schizophrenia positive symptoms often show up as hallucinations, delusional thinking, and disorganized speech. On the other hand, negative symptoms include the lack of emotional expression, low motivation, and limited speech. Language difficulties specify the cognitive symptoms in individuals suffering due to schizophrenia^5^. Further, schizophrenia patients are more vulnerable to suicide-related risks, with the lifetime suicidal rate of approximately 10%, which results in expensive medical treatments for patients^6,7^. For instance, Schizophrenia diagnosis and treatments are annually costing around 94 million Euros in Europe countries^8^.

Magnetic Resonance Imaging (MRI) scans, Electroencephalography (EEG), Positron Emission Tomography (PET) scans, and gene classification are widely used in the literature for the accurate diagnosis of Schizophrenia disorder^9^. Normally, psychiatrists detect schizophrenia disease by checking the presence of predefined symptoms through mutual interactions^9^. However, schizophrenia diagnosis might be misdiagnosed or unclear in many cases, since multiple comorbidities are involved in this disorder. Specifically, schizophrenia diagnosis is carried out using scan investigations, psycho-physio abilities, polygenic risk scores (PRS) and clinical factors^3^. Schizophrenia early-stage diagnosis is still evolving as a quite challenging problem in the medical domain, due to patient management and limited outcome issues^10,11^. Further, many of the schizophrenia patients are remaining untreated till today, due to crucial issues including social stigma and self-denial. Based on these aspects, the diagnosis of prodromal Schizophrenia symptoms is urgently needed, which can facilitate the treatment process and prevent the disease’s progression to severe stages.

Artificial Intelligence (AI) techniques are extensively used in the medical domain in the past decades, to enhance patient health statistics^12^. Specifically, Machine Learning (ML) and Deep Learning (DL) techniques are efficiently employed in the domain for the analysis of Bio-medical signals as well as medical images^12^. AI has opened up new frontiers in modern medicine, by generating possibilities for automating diagnoses, predicting disease severity, delivering medications with precision, personalizing treatments, and improving overall prognosis^13^. In the recent years, Explainable AI (XAI) has played a significant role in addressing challenges related to bias, safety and causality issues. Specifically, integrating AI-based frameworks with XAI for treatment or diagnostic purposes, significantly helps to improve transparency and reliability in diagnosis decisions predicted by the systems. Clinical validations of ML and DL models can be achieved with the the help of XAI architectures^14^. This study utilizes SHapley Additive exPlanations (SHAP) architecture^15^, with the game theory strategy and provides the feature importance-based ranking plots. Based on these aspects, the **primary contributions of this article** are listed as follows:

1. Assessment of crucial schizophrenia prodromal symptoms using Mutual Information (MI) based by Feature Importance computations.
2. Evaluation of Different ML classifiers such as Logistic Regression (LR), LR with GridSearchCV, Random Forest (RF), Decision Tree (DT), K-Nearest Neighbors (kNN), Support Vector Machine (SVM), Gradient Boosting (GB), XGBoost, Light Gradient Boosting Machine (LGBM) and Multilayer Perceptron (MLP).
3. Development of an enhanced and efficient classifier by employing **Ensemble Learning based STACK of meta-learners**.
4. Analysis of the proposed customized STACK models against existing frameworks for predicting schizophrenia symptoms in terms of various metrics including Accuracy, Precision, Recall, F1-score, ROC-AUC values and confusion matrices.
5. Integration of XAI layer in our classifier with **SHAP architecture using Violin, Force, Waterfall and Dependence plots**, so that clear insights and valuable interpretations of classifier predictions can be delivered.

The rest of this article is organized as follows: Section 2 describes literature review, Section 3 discusses materials and methods. A detailed result analysis is illustrated in Section 4, discussions and comparisons in Section 5 and the Section 6 includes conclusion and future scope.

## Literature review

In the literature of schizophrenia diagnosis, many research studies are attempted using MRI, PET, EEGs, psycho-physio investigations and gene classifications for detecting Schizophrenia disorder^3,11,18^. In the recent years, many researchers used popular ML techniques including SVM, RF, LR and Ridge classifiers for accurately predicting schizophrenia disease^7,9^. For instance, Shankar et al.^3^ explored multiple ML techniques, that are employed in schizophrenia diagnosis in a comprehensive manner. Lai et al.^4^ conducted a research survey on various ML methods used in schizophrenia detection and highlighted the potential of all those techniques. Santos et al.^5^ introduced a Multi-Kernel based Learning classifier and integrated Boruta feature selection approaches to identify the schizophrenia symptoms in patients. The event-related potential components collection of this framework is quite complicated and results in cognitive deficits in Schizophrenia patients. Kritiprasanna et al.^16^ presented a multichannel EEG signals based framework incorporating multivariate iterative filtering for schizophrenia prediction, which fails to focus more on physiological signals. Very recently, Sridevi and Shiny^9^ introduced a CNN-based multichannel EEGs framework for schizophrenia diagnosis. Geetha et al. used different neuroanatomical markers including cortical, subcortical volumes along with ensemble methods for schizophrenia prediction^17^. In the recent years, many researchers attempted on schizophrenia diagnosis using fMRI^18^, PET^19^, EEGs^16^ and a wider range of ML techniques including DT, LR, SVM and Neural Networks^4,20^ -^24^. Recent studies demonstrated the effectiveness of deep learning models such as CNNs and ResNet architectures for automatic feature extraction from EEG and neuroimaging data^25^ . Also, very recent hybrid deep learning frameworks, which combine temporal and functional features have shown improved diagnostic performance and robustness^26^. However, these approaches primarily rely on high-dimensional neuroimaging or EEG data, which require expensive acquisition, complex preprocessing, and limit real-world clinical applicability.

To summarize, while existing works demonstrate strong predictive performance using conventional ML models, they often (i) focus primarily on final diagnosis rather than early/prodromal stages, (ii) rely on single-model approaches with limited generalization, and (iii) lack interpretability features, which reduce their clinical applicability. Additionally, the limited integration of heterogeneous features and absence of explainable AI techniques could be considered as key gaps of existing literature. From another perspective, identifying biomarkers to predict schizophrenia prodromal symptoms is needed to speed-up the treatment process, so that the lifetime of patients can be saved. To address these issues, the proposed framework focuses on readily available clinical and behavioral features, which makes it more suitable for early-stage screening and deployment in resource-constrained settings. Further, unlike many deep learning models that operate as black boxes, the proposed method integrates ensemble learning with SHAP-based explainability and provides both competitive performance and interpretability, which is an essential requirement for clinical decision support.

## Methods and materials

### Dataset description

In this research study, a popular open-source dataset from Kaggle ’Schizophrenia Symptoms’ is employed, which includes data on 5000 patients with 10 attributes^27^. In this study, Kaggle dataset is selected due to its merits as given by: a) Open-access availability, b) Well-annotated labels and c) Widely used in multiple benchmark studies, which facilitates reproducibility and fair comparison with existing methods. Further, this dataset contains clinically relevant features that are associated with schizophrenia prodromal symptoms, which makes it suitable for developing and validating the proposed model. This multi-featured dataset consists of clinical, psychological and behavioural symptoms in patients. Specifically, in this study, prodromal symptoms refer to early behavioral and functional changes, such as psychomotor slowing, reduced self-care, decreased physical activity, and fatigue, which precede the clinical diagnosis of schizophrenia and are commonly reported in psychiatric literature as early indicators of schizophrenia progression. Since the dataset does not contain an explicit label for the prodromal phase, these symptoms are operationalized through relevant clinical variables available in the dataset. More specifically, the attributes including Slowing, Hygiene, Movement, Fatigue, and Pain represent measurable indicators associated with early neuropsychiatric and functional decline. These variables are recorded as normalized clinical scores (in -0.1 to 1.1 range), which reflect the observed intensity of each symptom. In this way, prodromal symptom patterns are implicitly captured by these multidimensional features and utilized by the model to assist in predicting the severity class of schizophrenia. The target variable ’Schizophrenia’ is represented in the form of 5-categories or classes of severity of disease as given by:

- **Class 1:** Low proneness
- **Class 2:** Moderate proneness
- **Class 3:** Elevated proneness
- **Class 4:** High proneness and
- **Class 5:** Very high proneness

Table 1 shows all the patient’s attributes and their descriptions used in this dataset.

**Table 1:** Description of attributes from Patient Dataset.

S.No	Attribute	Description
1	**Name**	Patient’s name, anonymized in real dataset
2	**Age**	Patient’s age varies between 55-95 years
3	**Gender**	Gender of patient
4	**Marital status**	Marital status of Individual
5	**Fatigue**	clinically observed degree of fatigue,
		indicated in -0.1 to 1.09 range
6	**Slowing**	Quantified Indicator of psychomotor slowing
		indicated in range of -0.09 to 1.09.
7	**Pain**	Intensity of experienced pain
		represented in -0.09 to 1.09 range
8	**Hygiene**	self-care or hygiene levels due to psychiatric
		disorders indicated in -0.08 to 1.08 range
9	**Movement**	Level/fluidity of physical movement indicating
		neuropsychiatric conditions in -0.09 to 1.09 range.
10	**Schizophrenia**	5-classes of severity diagnosis status

**Figure 1:**
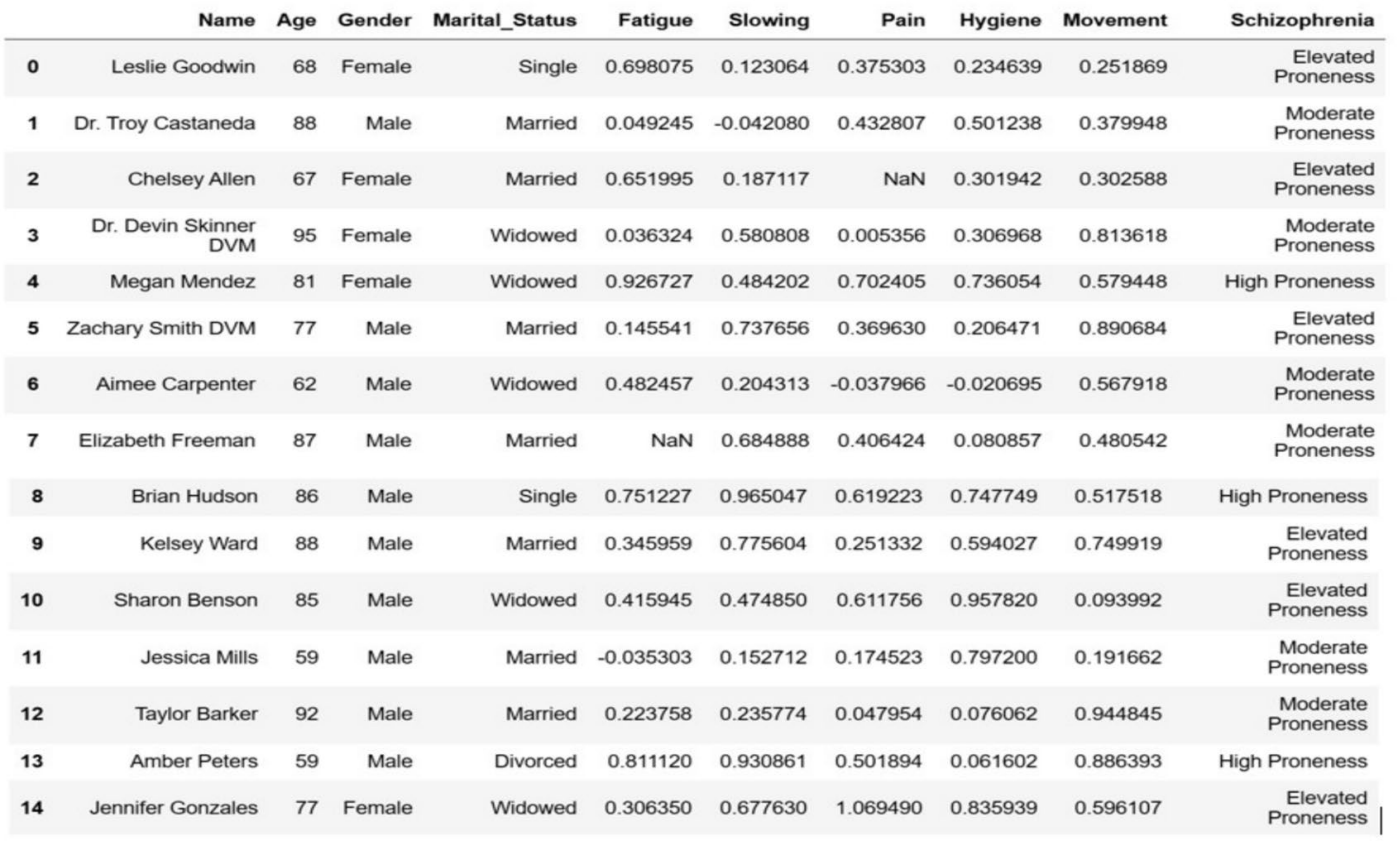
Sample snapshot of Patient Database.

Figure 1 presents the sample snapshot of the input patient’s DB, in which the attributes including Age, Gender, Marital status, Slowing, Hygiene, Movement, Pain, Fatigue and target feature Schizophrenia are represented in column-specific format.

### Data pre-processing

**Figure 2:**
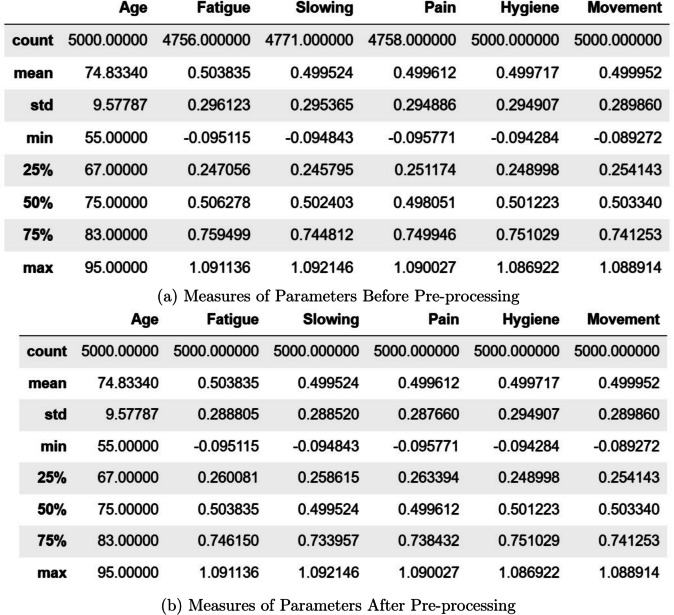
Descriptive Statistical Measures of parameters **a** Before and **b** After Pre-processing.

The experimental dataset includes 10 attributes: 5 Decimal, 4 String and 1 integer type. Specifically, the numerical attributes including Fatigue, Slowing, and Pain were consisting of 244, 229 and 242 null values respectively. These null entries are handled using Mean imputation strategy, in which each missing entry is replaced with the corresponding feature mean so that distributional consistency can be maintained and bias due to arbitrary replacement can be avoided. In addition, categorical attributes such as Gender and Marital Status are appropriately encoded into numerical representations for ensuring compatibility with ML models. All numerical features are further normalized to a common scale by aligning with their observed ranges to prevent dominance of any single feature during model training. Furthermore, Non-informative identifiers, including S.No and Name, are removed as part of feature selection since they do not contribute to predictive modelling. In this way, the overall preprocessing pipeline comprising missing value imputation, feature encoding, normalization, and feature selection is consistently applied prior to training all models to ensure reproducibility and fair evaluation of proposed model. Figure 2 illustrates the descriptive statistical measures of input parameters of the dataset before and after pre-processing.

**Figure 3:**
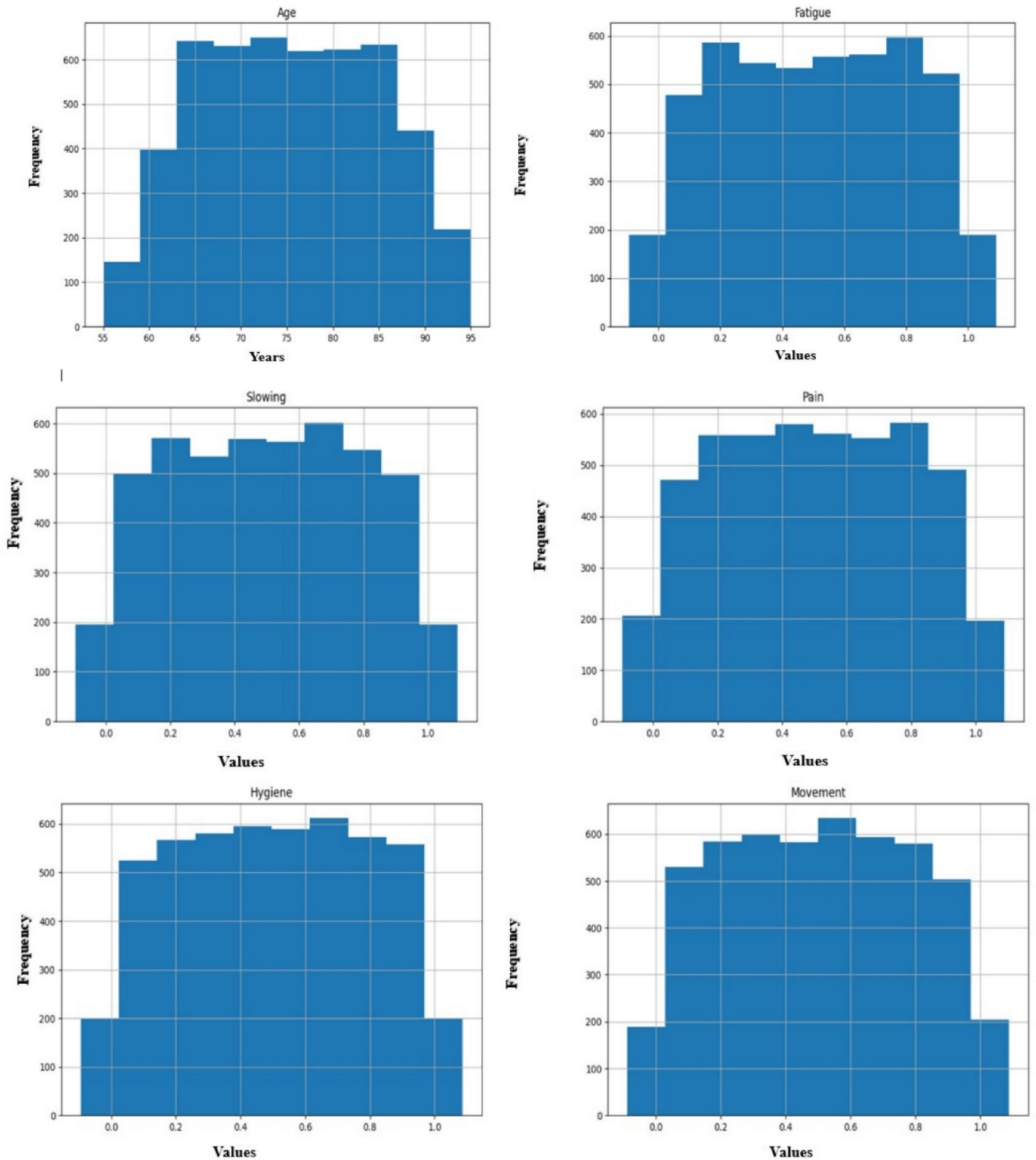
Histogram of input attributes of the dataset.

Figure 3 illustrates histogram of input attributes, in which X-axis indicates values of corresponding parameters and Y-axis represents frequencies of corresponding features.

**Figure 4:**
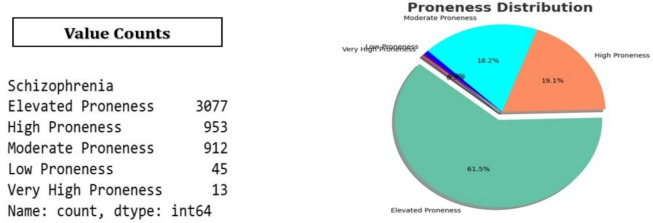
Proneness Distribution (Severity Class-wise).

The distribution of target variable ’Schizophrenia’ across 5-classes of proneness is illustrated in Fig. 4. Specifically, left side of Fig. 4 indicates the count of values/instances in each severity class, whereas right part of Fig. 4 shows the Proneness distribution in Pie-chart representation. As illustrated in Fig. 4, the target variable Schizophrenia is distributed across five severity classes. In this dataset, moderate class imbalance is observed, in which certain severity levels contain fewer instances compared to others. Such imbalance can bias the model toward majority classes, which might potentially affect the prediction performance for under-represented classes. To mitigate this issue, we ensured the use of evaluation metrics beyond accuracy, including precision, recall, F1-score, and AUC, which provide a more balanced assessment of model performance across all classes. Further, the ensemble stacking framework helps to improve generalization by combining diverse learners, and thereby reduces bias toward dominant classes.

**Figure 5:**
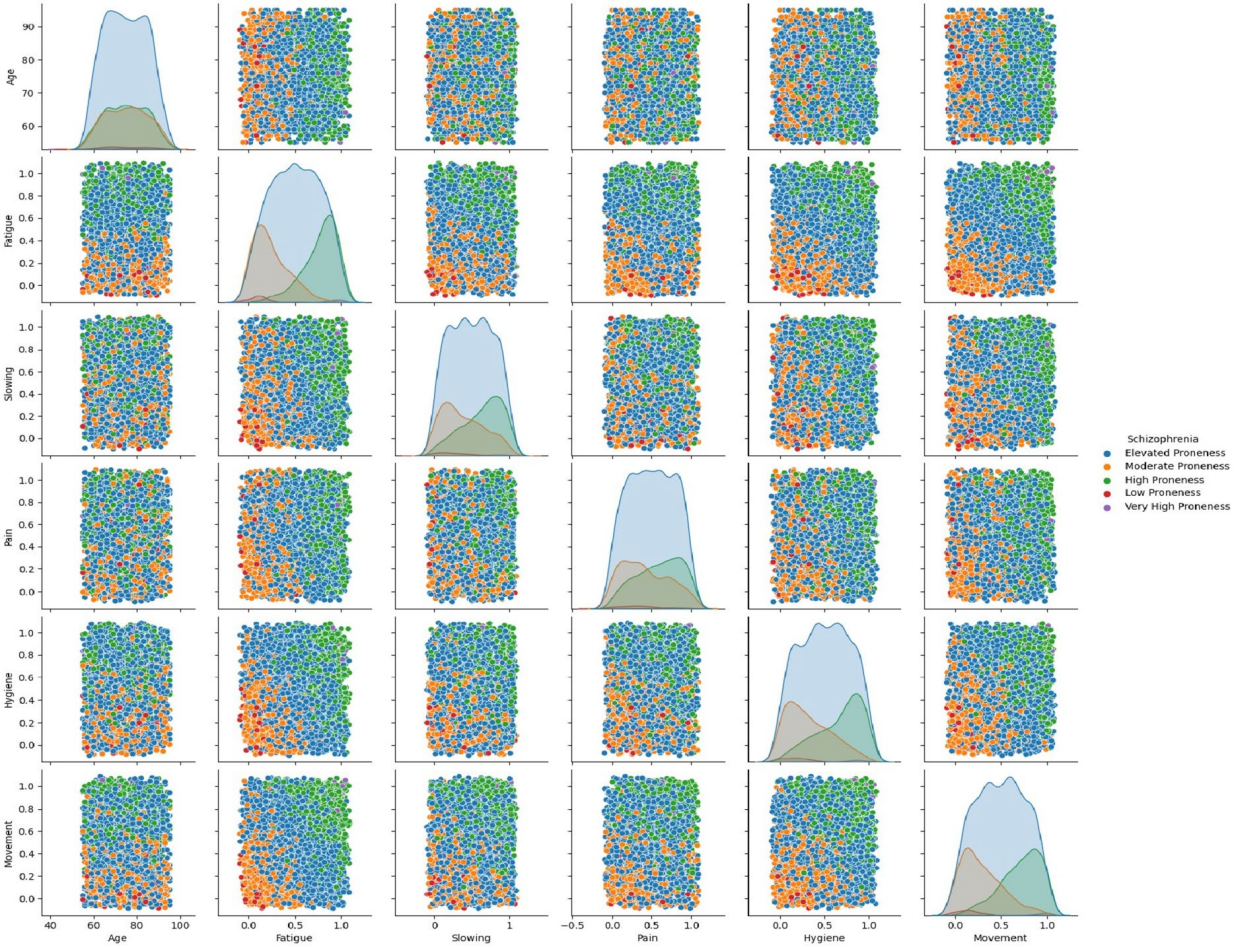
Pairplot showing pair-wise relationships, w.r.to target variable.

We used the python seaborn library to generate a Pairplot of input attributes, which clearly describes the pairwise relationships between the numerical parameters. Specifically, Fig. 5 represents the pair-wise relationships including the association with the target variable of the dataset by means of a grid of scatter plots.

## Results

### Performance metrics

The proposed models are evaluated and compared using standard classification performance metrics including Accuracy, Precision, Recall, F1-score, AUC-ROC score (Area Under the Receiver Operating Characteristics curve) and confusion matrices. The classifiers are designed to predict whether a specific patient exhibits symptoms of Schizophrenia at any of the 5-stages of severity level.

### Proposed Framework Evaluation

The proposed research study is evaluated and analysed using 10 individual Machine Learning and Deep Learning models as given by: Logistic Regression (LR), LR with GridSearchCV, Random Forest, Decision Tree (DT), K-Nearest Neighbors (kNN), Support Vector Machine (SVM), Gradient Boosting (GB), Light Gradient Boosting Machine (LGBM), XGBoost and Multilayer Perceptron (MLP). For the optimal selection of hyper-parameters GridSearchCV approach is employed. In this research study, Ensemble learning approach is utilized to build a distinct and efficient model for accurately predicting the schizophrenia disease.

In general, stacking algorithm combines the predictions of multiple base learners using a meta-learner and thereby creates the best-performing model for the given prediction task. In this study, the choice of ensemble stacking is motivated by both methodological and data-specific considerations as given by: Unlike bagging, which primarily reduces variance by training multiple instances of the same model on resampled data (e.g., Random Forest), and boosting, which sequentially focuses on correcting errors of weak learners (e.g., Gradient Boosting, XGBoost), stacking enables the integration of heterogeneous models and learns how to optimally combine their predictions through a meta-learner. In addition, in this study, the dataset comprises heterogeneous clinical features, where different model families (e.g., linear models like LR, kernel-based models like SVM, and non-linear models like MLP and tree-based methods) capture complementary decision patterns. Specifically, Stacking is preferred in this research study, due to its merits as given by:

- **Heterogeneity Handling:** It allows combining fundamentally different learners, unlike bagging which typically uses homogeneous models.
- **Meta-learning Capability:** The meta-learner in stacking learns optimal weighting and interactions among base model predictions, and thereby improves generalization.
- **Error Complementarity:** Instead of sequential error correction (like boosting), stacking employs diverse error distributions across models simultaneously.

**Figure 6:**
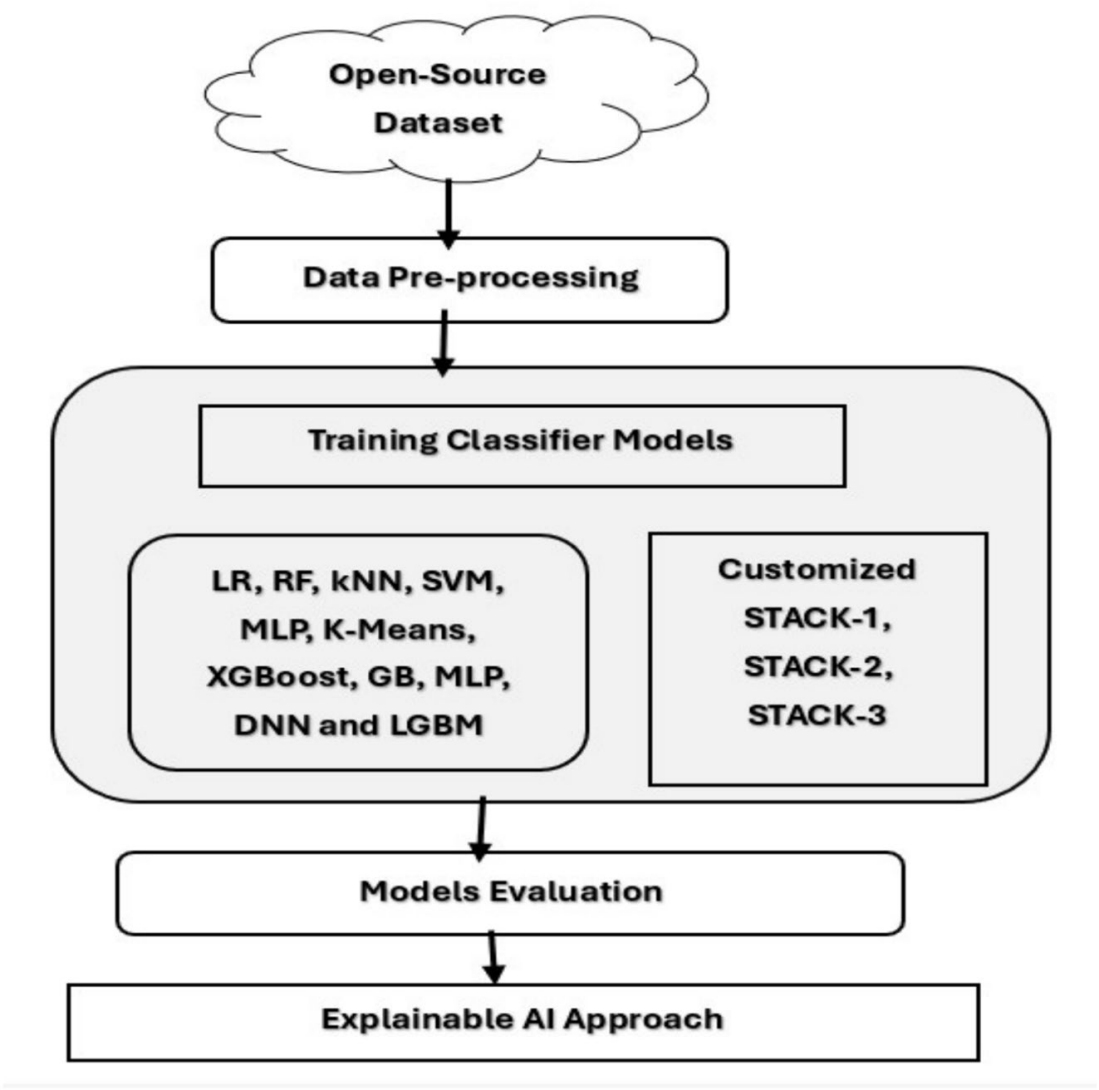
Architecture of the proposed framework.

**Figure 7:**
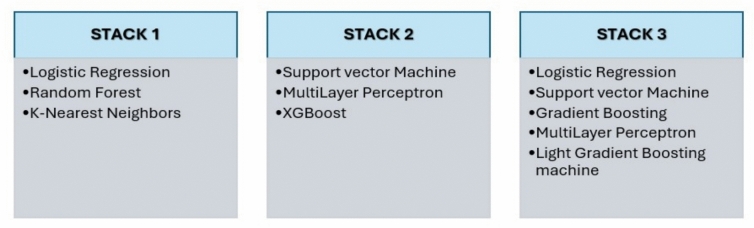
Description of customized STACK models.

Based on these aspects, we employed 3 stacking models in this schizophrenia prediction task. STACK-1 combined LR, RF and kNN classifiers and STACK-2 aggregated SVM, MLP and XGBoost classifiers. STACK-3 integrated LR, SVM, GB, MLP and LGBM classifiers for the prediction task. Figure 6 represents the overall architecture of the proposed framework. Figure 7 illustrates the pictorial representation of unique Ensemble models created in this study.

In this research study, an exhaustive validation is carried out by examining every possible combination of parameters to identify the optimal hyper-parameters of classifier models. The resultant optimal or best selected parameters for each classifier model, which are employed in this study are illustrated in Table 2 along with their corresponding descriptions.

**Table 2:** Description of best selected parameters for each Classifier model.

ML Classifier	Best Hyper-Parameters Specifications
**Random Forest**	{ ’min_ samples_ leaf’: 1,’n_ estimators’: 100,
	’criterion’: ’gini’, ’max_ features’: ’sqrt’,
	’min_ samples_ split’: 2, ’bootstrap’: True}
**Logistic Regression**	{’C’:1.0, ’penalty’:L2, ’max-iterations’:100,
	’Class-weight’:’Balanced’}
**LR-GridSearchCV**	{’C’: 10, ’solver’: ’saga’,’CV’:5}
**Decision Tree**	{ min_samples_leaf:1, ’criterion’:’gini’,
	’min_samples_split’:2, ’splitter’:’best’, }
**K-Nearest Neighbors**	{n_neighbors:3}
**Support Vector Machine**	{ ’gamma’: auto, ’kernel’: linear}
	’decision_function_shape’: ’ovr’,}
**Gradient Boosting**	{’n_estimators’:100, ’loss’:’log_loss’, ’learning_rate’:0.1,
	’criterion’:’friedman_mse’, ’subsample’:1.0,
	’min_samples_split’:2, ’max_depth’:3}
**Light Gradient Boosting**	{ ’num_leaves’:31, ’max_depth’:-1, ’boosting_ type’:’gbdt’,
**Machine**	’n_estimators’:100, ’learning_rate’:0.1,
	’subsample_for_bin’:200000,
	’min_child_ samples’:20, ’min_ child_ weight’:0.001}
**XGBoost**	{’booster’:’gbtree’, ’n_estimators’:100,
	’max_depth’:6, ’learning_rate’:0.3, ’min_child_weight’:1,
	’colsample_bytree’:1, ’eval_metric’:’mlogloss’}
**Multilayer Perceptron**	{’activation’:’relu’, ’hidden_layer_sizes’:100,
	’batch_size’:200, ’solver’:’adam’,’alpha’:0.0001,
	’max_iter’:500’, learning_rate_init’:0.001, }

Table 3 illustrates the performance results of all 10 machine learning models on 80:20 data split ratio, by means of Accuracy, Precision, Recall, F1-score and Average AUC scores. Optimized LR and MLP techniques are achieving higher scores when compared to other ML methods.

**Table 3:** Performance of 10 ML Classifier Models using 80:20 data split ratio.

ML Classifier	Accuracy	Precision	Recall	F1-Score	Average
	(in %)				**AUC**
**Random Forest**	88.93	0.91	0.89	0.90	0.7475
**Logistic Regression**	93.2	0.92	0.93	0.93	0.7552
**LR-GridSearchCV**	**95.6**	0.91	0.89	0.90	0.7918
**Decision Tree**	81.33	0.82	0.81	0.81	0.7739
**K-Nearest Neighbors**	87.53	0.88	0.88	0.87	0.7768
**Support Vector Machine**	93.93	0.94	0.93	0.94	0.7882
**Gradient Boosting**	89.8	0.90	0.89	0.90	0.7737
**Light Gradient**	90.2	0.90	0.90	0.90	0.7941
**Boosting Machine**					
**XGBoost**	90.1	0.90	0.90	0.90	0.8147
**Multilayer Perceptron**	**95.4**	0.95	0.95	0.94	0.8514

Table 4 presents the performance results of all the 3 customized STACK ensemble learning models, which are developed in this research study. It can be observed from Table 4 results that, STACK-3 is performing superior when compared with other 2 stacking classifiers and 10 ML models in terms of achieving higher accuracy, precision, recall, F1-score and AUC values.

**Table 4:** Performance of Customized Stack Ensemble Learner Models.

Classifier	Accuracy	Precision	Recall	F1-Score	Average
	(in %)				**AUC**
**STACK-1**	95.66	0.95	0.96	0.95	0.92
**STACK-2**	96.21	0.96	0.95	0.961	0.93
**STACK-3**	**96.26**	0.96	0.96	0.967	0.93

**Table 5:** Class-Specific Performance Results of 3 STACK Models.

Model	Precision	Recall	F1-Score	Support	AUC
**STACK-1**					
**Class 1:**	0.97	0.98	0.97	945	0.96
**Class 2:**	0.94	0.94	0.94	244	0.96
**Class 3:**	1.00	0.38	0.56	13	0.69
**Class 4:**	0.94	0.94	0.94	293	0.96
**Class 5:**	0.00	0.00	0.00	5	0.50
**STACK-2**					
**Class 1:**	0.97	0.98	0.97	945	0.96
**Class 2:**	0.95	0.94	0.94	244	0.97
**Class 3:**	1.00	0.69	0.82	13	0.85
**Class 4:**	0.95	0.95	0.95	293	0.97
**Class 5:**	1.00	0.80	0.87	5	0.90
**STACK-3**					
**Class 1:**	0.97	0.98	0.97	945	0.96
**Class 2:**	0.95	0.95	0.95	244	0.97
**Class 3:**	1.00	0.62	0.76	13	0.85
**Class 4:**	0.95	0.95	0.95	293	0.97
**Class 5:**	1.00	0.82	0.89	5	0.90

This research study involves multi-class classification of schizophrenia disorder, we aim to evaluate the effectiveness of each stacked model using class-specific performance metrics. Table 5 illustrates the class-specific performance results of all the 3 stack models developed in this study. Specifically, each class-specific results highlight the effectiveness of each model in accurately predicting the severity of the schizophrenia disorder.(i.e. class 1: Low proneness and so on). It is evident from Table 5 that, STACK-3 is outperforms the other 2 stacking classifiers in terms of scoring higher precision, recall, F1-score and AUC values based on class-specific performance metrics.

**Figure 8:**
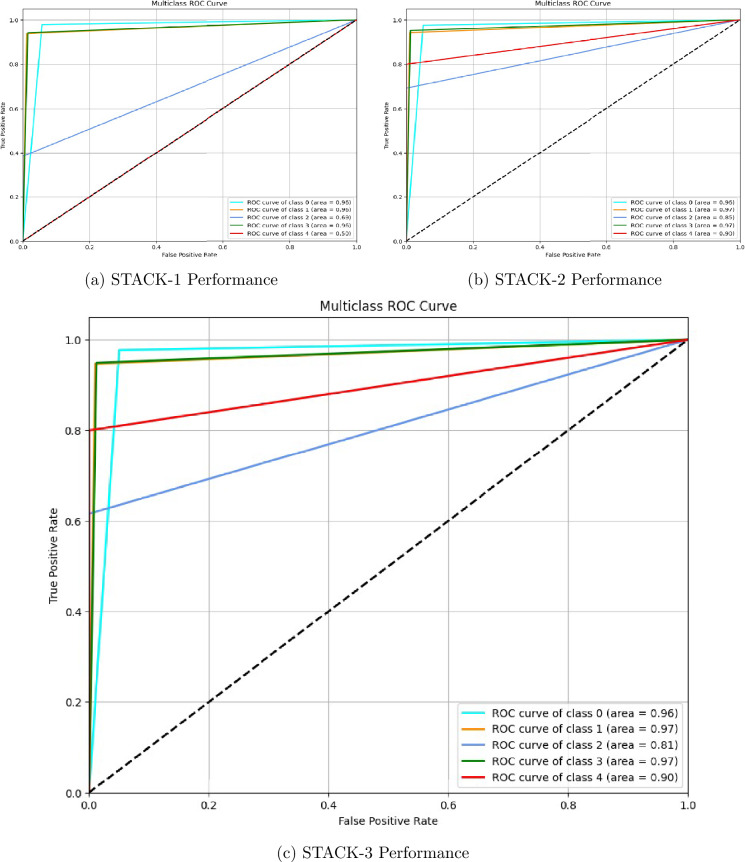
Performances results of 3 STACK Models using ROC Curves & AUC values.

**Figure 9:**
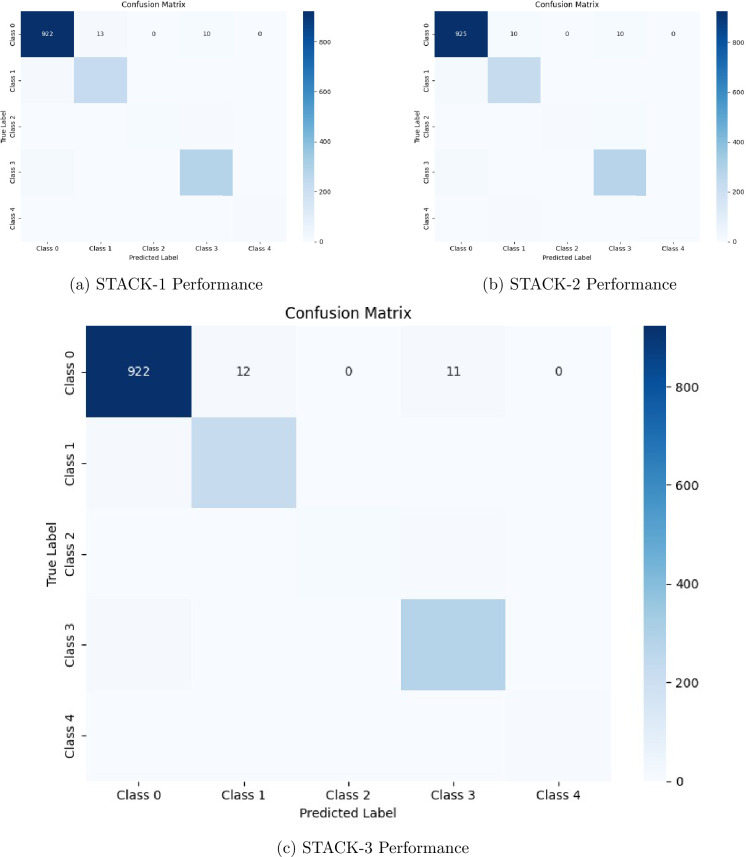
Confusion Matrix showing Performance Evaluation of 3 STACK Models.

Figure 8 describes the performance results of all the three STACK classifiers using ROC curves and AUC values (indicated at the bottom of plot) of each severity class in Schizophrenia detection. It can be observed from Fig. 8 plots that, STACK-3 is scoring superior results in terms better multi-class ROC curves and AUC values, when compared to other two STACK models. Figure 9 represents the performance evaluation of proposed 3 STACK models using confusion matrices, in which STACK-3 classifier is achieving slightly better scores when compared to the other 2 STACK models.

### Feature importance with mutual information

**Figure 10:**
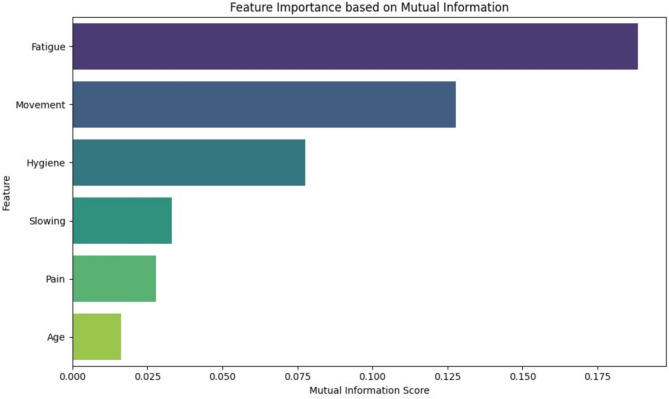
Feature importance with mutual information.

Mutual Information (MI) algorithm is a popularly known filter technique of feature selection, which considers the statistical properties of the input attributes. MI algorithm uses entropy measure, which measures the variability in the input features. If the Mutual information value of 2 attributes is very high, then it implies that they are strongly related, and if the value is zero then, the features are completely unrelated. We applied this Mutual Information algorithm to the Schizophrenia dataset, which clearly indicates how strongly the individual features are related to the target. Figure 10 illustrates the Feature Importance with MI plot, in which the input attributes are ranked as per their individual contribution towards the target variable. From Fig. 10 it is evident that, Fatigue, Movement attributes are strongly related in this schizophrenia prediction task, when compared with the remaining features of patients.

### Explainable AI (XAI) interpretations used in study

This section illustrates several XAI interpretations utilized in this study, for accurately explain the predictions and to interpret the classifier behaviours. We used different visualization plots generated from the Shapley Additive exPlanations (SHAP) architecture in this study. The visualization plots used in this study helped to clearly justify the model predictions and thereby to understand the decision-making process of the classifiers. In the SHAP architecture, each feature is assigned an ‘importance value’, which clearly measures the contributions of the respective attribute in this schizophrenia prediction task. The SHAP-visualization plots provide valuable and meaningful insights into the schizophrenia disease predictions generated by the classifiers. In this aspect, we utilized different visualizations including Violin, Waterfall, Force and Dependence plots to effectively interpret the classifier predictions related to schizophrenia severity category. In this study, LR-GridSearchCV was one of the best performing ML meta-learners with an accuracy and precision of 95.6% and 91%, respectively. We employed this model to demystify its schizophrenia predictions using SHAP visualization plots.

#### SHAP violin plot

**Figure 11:**
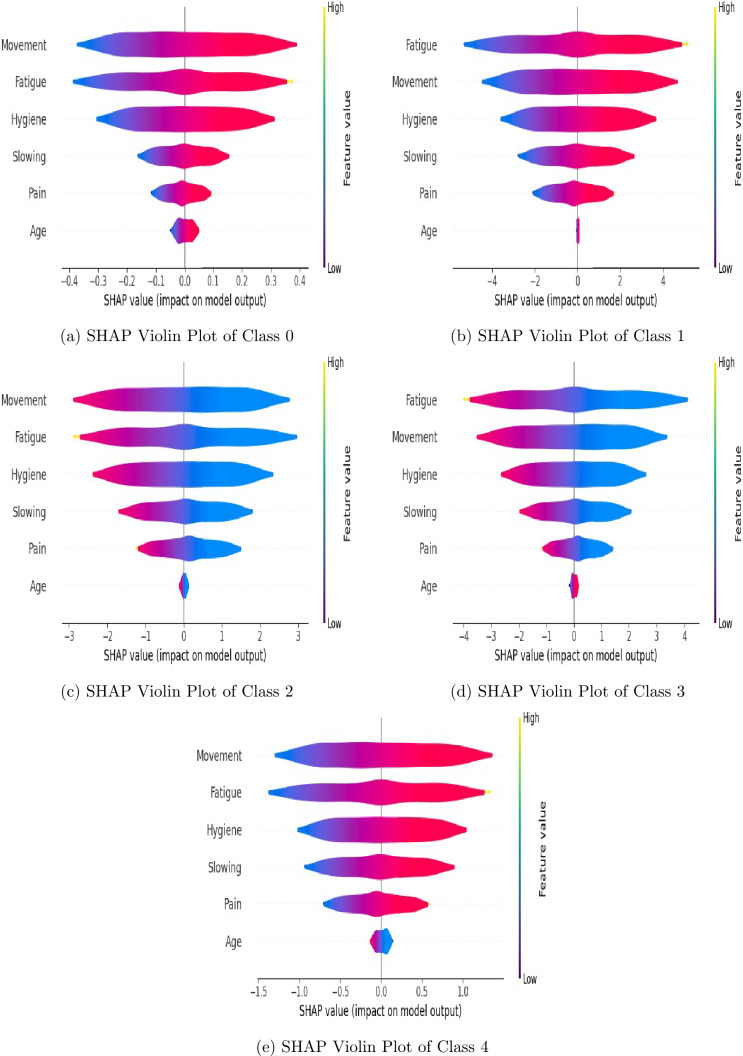
SHAP Violin Plots Illustrating Feature Impact Across All Five Schizophrenia Severity Levels **a** Class 0, **b** Class 1, **c** Class 2, **d** Class 3 and **e** Class 4.

A SHAP Violin plot is created for one of the best-performing linear model in this study - Logistic Regression. In general, SHAP works particularly well with linear models such as Logistic Regression, since its additive nature of values aligns naturally with the predictions of these models. Figure 11 shows the SHAP Violin plots generated for each severity class of schizophrenia disease. As indicated in Fig. 11, the horizontal axis indicates SHAP values, and the color indicates the lower or higher value of the data points. The input features are organized as per their importance, and the best attributes are appearing on the top. Bright pinkish red indicates a higher feature value, whereas bright blue/purple indicates a lower feature value. In Fig. 11 Violin plots of all 5 classes, it is evident that the most crucial feature is ’Fatigue’, which indicates higher values of Fatigue (Yellow dots on the right) represents higher likelihood of very high proneness of schizophrenia symptoms. It is revealed that schizophrenia prediction of LR is based on the patients having severe Fatigue, Movement and Hygiene symptoms. From Fig. 11, it is evident that, Violin plot interpretations reasonably agrees with the conventional symptoms of the schizophrenia disease such as Age attribute minimally contributes to the prediction.

#### SHAP waterfall plot

**Figure 12:**
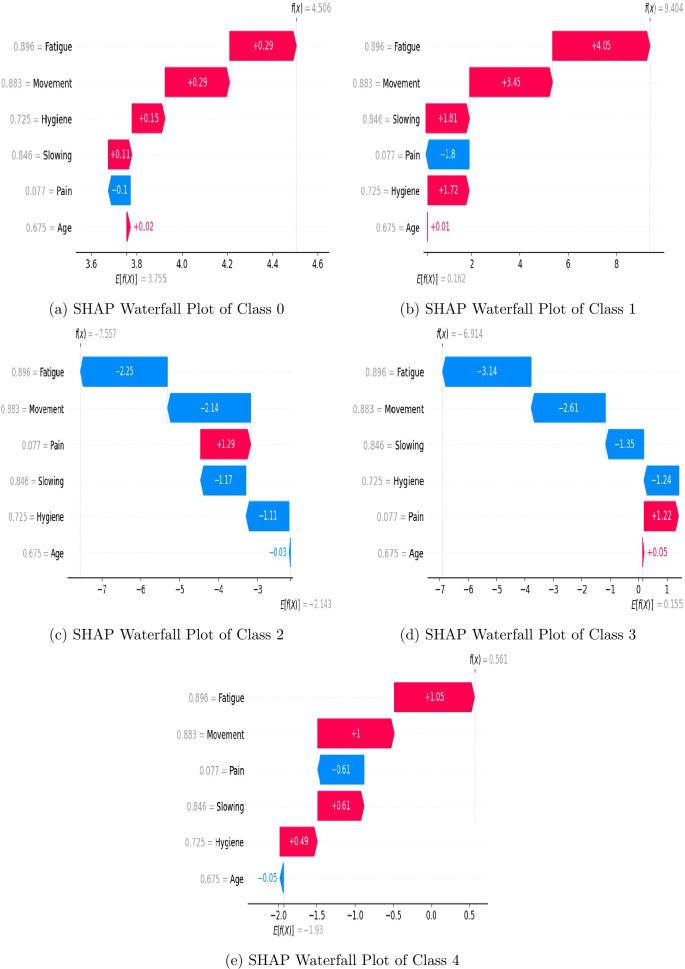
SHAP Waterfall Plot of 5 Severity Classes of Schizophrenia.

Figure 12 shows the SHAP Waterfall plots generated in this study, for interpreting all the 5 severity classes of Schizophrenia disorder. In each severity class’s waterfall plot, the horizontal axis indicates the expected values of the classifier output and right or left shifts emphasizes the positive or negative contribution of individual patient’s attributes. Random 5 patient’s records are considered for analysis such that these patient’s are diagnosed with 5 severity classes of schizophrenia disorder as per dataset. The feature values of the considered data record are shown in Grey color along the y-axis, which are organized from most to least important in top to bottom fashion. In waterfall plot of each class, the SHAP values describe the difference between the prediction and the expected values as given by:

1 $$\begin{aligned} f(x) = \mathbb {E}[f(x)] + \sum _{i=1}^{n} \phi _i \end{aligned}$$

where, *f*(*x*) indicates the model’s prediction for a given instance, *E*[*f*(*x*)] represents the average model output over the dataset, $\phi _i$ indicates the SHAP value for feature, in terms of its contribution to the prediction and *n* represents number of input features respectively. It can evident from the Fig. 12 that, waterfall plots of all 5 severity classes are almost flat with small jumps, indicating no features are strongly driving the prediction in either ways. The plots of Class 0, Class 1 and Class 4 emphasize strong positive evidences from features as $f(x)>\mathbb {E}[f(x)]$, whereas Class 2 and Class 3 highlight strong negative evidences from features as $f(x)<\mathbb {E}[f(x)]$. Fatigue and Movement are the most impactful features as indicated in Fig. 12. In this way, Fig. 12 waterfall plots facilitates the visualization of the shifts of SHAP values from the prior expectations to till the final prediction.

#### SHAP force plot

**Figure 13:**
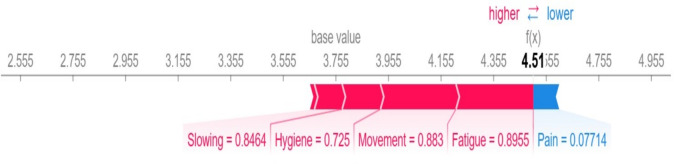
SHAP Force plot.

Figure 13 shows the SHAP Force plot, which facilitates the end-users to accurately determine the most crucial features involved in prediction for a given patient record. Higher scores shown in red, leads to high proneness predictions and the lower scores, shown in blue, leads to low proneness predictions. In SHAP Force plots, most crucial features on prediction appear close to dividing boundary and the bar size measures the influence of the given attribute. As seen in Fig. 13, Fatigue is the most significant feature in predicting very high proneness for the randomly selected patient sample.

#### SHAP dependence plot

**Figure 14:**
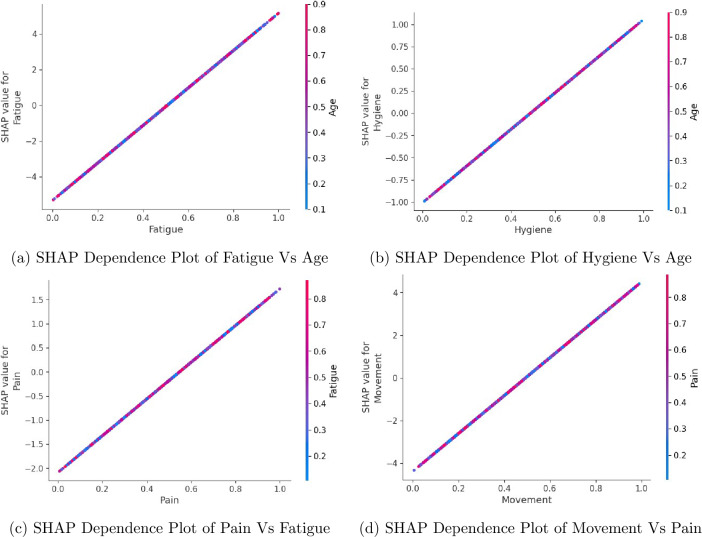
SHAP Dependence plots indicating **a** plot of Fatigue against Age **b** Plot of Hygiene against Age **c** plot of Pain against Fatigue **d** Plot of Movement against Pain.

SHAP Dependence plot shown in Fig. 14, indicates how the classifier predictions depends on the input feature, where each datapoint on the plot indicates one patient. In Fig. 14 plots, x-axis indicates the attribute value whereas y-axis represents the SHAP values and the color represents to the second feature. Figure 14 four plots, illustrate the interaction between Fatigue, Age, Pain, Hygiene and Movement features. We used linear model for this plots this SHAP visualizations and the SHAP value is changing linearly with the feature values. In this way, SHAP dependence plots of different features, helped to obtain findings about feature interactions as well as their effects on the given Schizophrenia prediction task.

In the proposed framework, SHAP-based XAI models contribute to clinical decision-making in the following ways:

- **Patient-Specific Risk Interpretation**: SHAP force and waterfall plots provide instance-level explanations by quantifying how individual features (e.g., Slowing, Hygiene, Fatigue) contribute to a specific prediction. This enables clinicians to identify which symptoms are driving the model’s decision for a given patient, and thereby supports personalized assessment rather than relying solely on a predicted class label.
- **Clinical Feature Prioritization/Global Insights**: SHAP summary and dependence plots highlight the most influential features across the population, and thereby help clinicians to understand key prodromal indicators that are associated with schizophrenia severity. This can guide focused clinical evaluation and monitoring strategies.
- **Transparency and Trust in AI-Assisted Decisions**: By explicitly showing feature contributions, SHAP reduces the “black-box” nature of the model, and thereby allows clinicians to validate whether predictions align with established clinical knowledge, which is critical for adoption in real-world healthcare settings.
- **Decision Support, Not Replacement**: The SHAP explanations are designed to complement clinical judgment by providing evidence-backed reasoning for predictions, and thereby support more informed and confident decision-making rather than serving as stand-alone visual outputs.

### Comparison of proposed vs existing models

Multiple research studies utilized machine learning models for schizophrenia prediction using open-source datasets such as Kaggle dataset, which is utilized in this study. Anchal et al.^20^ used DT, SVM, NN based classifiers and SVM, GB based ensemble models for the schizophrenia prediction task, which achieve slightly lesser scores, when compared with the proposed study, as shown in Table 6. Shravan et al.^21^, sachin et al.^22^ and Swathi et al.^23^ used different ML techniques such as RF, SM, DL and LR and showed better performance in the schizophrenia symptoms detection task, as shown in Table 6. Most of the studies are mentioning RF and LR as the best-performing classifiers in the prediction task. However, our LR- based Ensemble model outperforms these architectures with an accuracy of 96.7%. Overall, the proposed customized ensemble learning based STACK models perform better compared to other studies in the literature. Further, XAI architectures such as SHAP visualizations are not used by other research studies for this schizophrenia prediction task. Instead of obscured black box predictions, we trust that, interpretable and reliable models would be more beneficial for real-world implementation in healthcare environments. Keeping this motivation in mind, we generated the proposed architecture along with meaningful visualizations, so that contributions towards de-mystifying the AI technology in healthcare can be successfully achieved.

**Table 6:** Comparison of Proposed Vs Existing Prediction Models.

S.No	Model	Classifier	Accuracy	Explainable
				**AI Techniques**
1	**[20]**	DT, SVM, NN	89.6%	-
2	**[21]**	SVM, RF, GB,LR	96%	-
3	**[22]**	DCNN, SVR, ViT	87%	-
4	**[23]**	RF, DL	96%	-
5	**Proposed**	RF, LR, DT, SVM, GB,	**96.7**%	SHAP (Violin, Waterfall,
	**Model**	XGBoost, MLP, kNN,		Force, Dependence) Plots
		LGBM		

## Discussion

Schizophrenia is a serious psychiatric disorder, which is associated with hallucinations, delusions, dangerous disorganized behaviours, cognitive impairments and social disengagements in patients. Most cases of schizophrenia disorder are remaining untreated till now, due to crucial factors such as failure to diagnose and self-denial. Based on these aspects, the early prediction of severity levels of schizophrenia symptoms is needed, which significantly facilitates the treatment process and also prevents the disease progression to more advanced stages. We developed 10 different ML and DL models in this research study and analysed their performance to identify the best-performing classifier for this prediction task.

All the ML frameworks are evaluated by considering the Best-parameters specifications as shown in Table 2 with the data split ratio of 80:20. This 80:20 data split ratio is selected after running numerous trails with other ratios such as 70:30 and 60:40. All the ML classifiers are giving comparable performances in this data split ration, which can be observed in Table 3. LR, MLP and SVM models are achieving slightly higher accuracy and specificity scores when compared with the other classifiers. In this multi-class classification task, we analysed the Class-specific performances of all the 3 STACK ensemble models to highlight their effectiveness in this prediction task, as tabulated in Table 5.

stacking models are employed in this study, which combine multiple base learners using a meta-learner for generating the best-performing ensemble model for this prediction task. The customized 3 STACK models are indicated in Fig. 7 and the performances of individual STACK models are illustrated in Table 4. STACK-2 and STACK-3 models employing LR, MLP, SVM classifiers are providing almost similar results, whereas STACK-3 is scoring slightly higher accuracy values. Table 4 performance results suggests that, the proposed meta-learners could serve as the better options, while developing a real-time Ensemble learning based Schizophrenia prediction systems. The proposed top-performing customized stack models noticeably decreased the no. of false negatives schizophrenia predictions, with only 18 out of 943 samples). We used Mutual Information(MI)-based feature selection, which gives useful insights about feature importance needed in training the classifiers. It is visible in Fig. 10 that, Fatigue and Movement attributes are significantly impacting features in this schizophrenia prediction task, when compared with others.

Further, in this study, we incorporated one layer of Explainable AI approaches to the top-performing meta-learner model. We investigated several types of SHAP plots to describe the LR model predictions based on feature importance. Specifically, SHAP Violin plot in Fig. 11 agree with the classic symptoms of the schizophrenia disease, by highlighting Fatigue and Movement are the crucial prodromal symptoms in schizophrenia patients. Figure 12 SHAP Waterfall plots emphasize strong positive as well as negative evidences from features of different severity classes. SHAP Force plot shown in Fig. 13, clearly highlights the crucial feature contributed in predicting high proneness schizophrenia in the given patient sample. We analysed feature interactions and their impact on this prediction task by considering features including Fatigue, pain and Age, as illustrated in Fig. 14. Notably all the types of SHAP plots described the predictions and emphasized Fatigue and Movement as highest significant features in this schizophrenia symptoms prediction task. Existing research on schizophrenia disease has indicated the importance of these features in diagnosis. In this way, XAI tools help to visualize and meaningfully interpret predictions, which can be considered by practitioners during the prediction of prodromal symptoms of schizophrenia disease.

The proposed framework differs from existing ML and ensemble learning (EL) approaches in several key aspects beyond incremental performance improvement, as given by:

- **Prodromal-Oriented Modeling:** Unlike prior studies that primarily focus on binary or multi-class schizophrenia diagnosis, the proposed work emphasizes early-stage (prodromal) symptoms modeling by employing clinically relevant behavioral and functional indicators (including Slowing, Hygiene, Movement, Fatigue). This enables the framework to capture subtle pre-diagnostic patterns rather than only final disease labels.
- **Customized Stacked Ensemble Architecture:** While existing studies commonly employ standalone models such as Random Forest (RF), Logistic Regression (LR) and basic ensembles, this work introduces the multi-level stacked ensemble framework, in which heterogeneous base learners are combined with the meta-learner to improve generalization. This design is specifically optimized for the given clinical feature space compared to the standard ensembling techniques.
- **Integration of Explainable AI (XAI):** Existing studies mainly depend on black-box predictions, whereas the proposed framework incorporates SHAP-based interpretability including force, waterfall, violin, and dependence plots. This provides both global and local explanations, and thereby facilitates clinicians to understand feature-level contributions to predictions, which is unexplored in present schizophrenia prediction studies.
- **Quantitative and Interpretability Trade-off:** This framework is designed to jointly optimize predictive performance and clinical interpretability in terms of achieving competitive accuracy (96.7%) while maintaining transparency. Existing works often prioritize accuracy alone without addressing clinical usability.
- **Operationalization of Clinical Features:** This study explicitly maps dataset attributes to clinically meaningful symptom constructs including psychomotor slowing and bridges the gap between data-driven modeling and psychiatric relevance, which is less focused in existing ML-based approaches.

## Conclusions and future scope

This study demonstrated an Ensemble Learning based framework with feature importance for accurately predicting the prodromal symptoms in Schizophrenia patients. The proposed STACK-3 classifier performed best with accuracy, precision, recall, F1-score and Average AUC of 96.2%, 96%, 96%, 96.7%, and 93% scores respectively. We utilized SHAP visualizations including Violin, Waterfall, Force and Dependence Plots, for providing valuable interpretations of our classifier predictions. However, focusing primarily on clinical, psychological, and behavioral features introduces certain limitations: a) First, such features are often subjective and observer-dependent, which may introduce variability and potential bias compared to objective biomarkers; b) Second, the absence of biological or neuroimaging data such as MRI, fMRI and genetic markers limits the ability to capture underlying neurophysiological mechanisms associated with schizophrenia, which could enhance early detection accuracy; c) Third, behavioral indicators may reflect overlapping symptoms across multiple psychiatric conditions, which potentially affect specificity. However, the features utilized in this study are is intentional, are non-invasive, cost-effective, and readily available in real-world clinical settings, which makes the proposed framework more practical for large-scale and early-stage screening applications in resource-constrained environments. Further the limitations of this current study are given by: (i) reliance on clinical, behavioral, and psychological features without incorporation of biological or neuroimaging data, (ii) potential subjectivity and variability in clinically observed attributes, (iii) use of a single dataset which may limit generalizability, and (iv) absence of explicitly labelled prodromal-stage annotations.

Future work will focus on multimodal integration, by combining clinical features with neuroimaging and biological data to improve robustness, diagnostic precision, and generalizability of the prediction model. Although, the Kaggle dataset provides a standardized evaluation platform, it may not fully capture the diversity and heterogeneity of real-world clinical data. This limitation can be addressed by conducting future validations on multi-center clinical datasets to establish the generalizability and practical applicability of the proposed framework. Further, this research study can be extended by integrating Web-based UIs for patient questionnaires and Mobile Applications for assessing patient’s daily activities and so on, which can assist the doctors and specialists in clinical decision-making process.

## Data Availability

Publicly available datasets were analysed in this study. The data supporting the findings of this study are available from the corresponding author upon reasonable request.
